# Clustering of cognitive subtypes in schizophrenia patients and their siblings: relationship with regional brain volumes

**DOI:** 10.1038/s41537-022-00242-y

**Published:** 2022-05-09

**Authors:** Erkan Alkan, Simon L. Evans

**Affiliations:** Faculty of Health and Medical Sciences, University of Surrey, Guildford, Surrey United Kingdom

**Keywords:** Schizophrenia, Biomarkers

## Abstract

Schizophrenia patients (SZH) often show impaired cognition and reduced brain structural volumes; these deficits are also detectable in healthy relatives of SZH. However, there is considerable heterogeneity: a sizable percentage of SZH are relatively cognitively intact; clustering strategies have proved useful for categorising into cognitive subgroups. We used a clustering strategy to investigate relationships between subgroup assignment and brain volumes, in 102 SZH (*N* = 102) and 32 siblings of SZH (SZH-SIB), alongside 92 controls (CON) and 48 of their siblings. SZH had poorer performance in all cognitive domains, and smaller brain volumes within prefrontal and temporal regions compared to controls. We identified three distinct cognitive clusters (‘neuropsychologically normal’, ‘intermediate’, ‘cognitively impaired’) based on age- and gender-adjusted cognitive domain scores. The majority of SZH (60.8%) were assigned to the cognitively impaired cluster, while the majority of SZH-SIB (65.6%) were placed in the intermediate cluster. Greater right middle temporal volume distinguished the normal cluster from the more impaired clusters. Importantly, the observed brain volume differences between SZH and controls disappeared after adjustment for cluster assignment. This suggests an intimate link between cognitive performance levels and regional brain volume differences in SZH. This highlights the importance of accounting for heterogeneity in cognitive performance within SZH populations when attempting to characterise the brain structural abnormalities associated with the disease.

## Introduction

Positive and negative symptoms constitute the primary diagnostic criteria for schizophrenia, but cognitive deficits are also one of the hallmark features of SZH. These are present in 75–80% of patients, impacts on daily functioning^1,2^, are present at onset and remain relatively stable over the course of the illness^3^. Unfortunately, cognitive impairments are largely unresponsive to pharmacological therapy^4^. Cognitive impairment in SZH has been demonstrated across almost all cognitive domains, most notably executive function^5^, processing speed^6^, attention and vigilance^7^, and working memory^8^. Cognitive training has limited efficacy, although there is some evidence of benefit for executive function training, for example^9^. Since deficits in cognitive abilities in SZH are largely independent of clinical state and medication status, they have been proposed as a potential endophenotypic marker for SZH^10^. Further, they are familial in nature. Meta-analyses indicate impairments in unaffected first-degree relatives versus controls across a variety of cognitive tasks and particularly those tapping executive function^11^. This points to a genetic overlap between cognitive deficits and risk for SZH; polygenic schizophrenia risk scores have been associated with lower general cognitive ability^10,12^, supporting this.

Nevertheless, not all SZH show cognitive impairment. A cognitively relatively intact subgroup of SZH seems to exist with up to 25% of patients showing minimal evidence of impairment; also, there is significant heterogeneity in severity across those that are impaired^13^. Recently, studies have characterised this heterogeneity by implementing clustering strategies to identify subgroups of SZH patients based on cognitive function abilities^13–18^; this approach has also been utilised in first episode patients^19,20^. Cluster analysis allows the classification of individuals into subgroups based on their cognitive profiles, and such analyses have tended to identify 3 distinct subgroups: a relatively intact group, an intermediate group (a subgroup with the level of cognitive deficits sitting between normal performance and severe cognitive impairment), and a severely impaired group showing global deficits^16,17^. Although not all studies have identified a relatively intact cluster group^21^, a recent meta-analysis of relevant studies confirmed that a 3-cluster solution is the most supported outcome (17 of 22 studies reviewed)^22^. A very limited subset of such studies has included neuroimaging data alongside the cognitive assessments, to examine the relationships between group assignment and brain structure, and these have shown links to the underlying neurobiology. The intact subgroup has less pronounced cortical thinning^23,24^; conversely, clustering patients according to cortical thickness patterns differentiates those with greater impairment^25^. On brain volumes, Weinberg, et al.^26^ found widespread reductions (including significantly reduced total hippocampal, insula, superior temporal sulcus, and frontal) in gray matter volumes in the impaired cluster compared to controls. In contrast, patients in the relatively intact group differed from controls only on inferior parietal volume, whereas the intermediate group had reduced inferior parietal and insula volumes. Only one study has extended the clustering approach to include first-degree relatives^15^. Across their whole sample of SZH, relatives, and controls, a 3-factor solution was found. Cognitive performance in first-degree relatives, while impaired relative to controls, is not as impaired as that seen in SZH^27^; accordingly, more than half of the relatives were assigned to the ‘intermediate’ cluster. Between-cluster differences in brain volumes were identified in frontal, temporal, and limbic regions; amongst these, Ohi, et al.^15^ highlighted anterior cingulate cortex (ACC) volume as an important between-cluster factor since it was largely independent of diagnostic status. However, the Ohi, et al.^15^ study was limited by fairly small groups of relatives (*N* = 20) and controls (*N* = 25); also, by including any first-degree relative, the relatives were significantly older than the other groups as more than half were parents.

The aim of this study was to provide further insights into cognitive subtypes within SZH and their first-degree relatives, and the relationship between subgroup assignment and brain volumes. The study expands upon and addresses shortcomings in the very limited pre-existing literature by utilising a better-matched and larger sample. For the relatives, only SZH-SIB were recruited, and a group of control siblings (CON-SIB) was also utilised to ensure ages were matched. We examined cognitive profiles of SZH and their siblings in comparison to age-matched control groups and then performed a cluster analysis on the cognitive parameters irrespective of the diagnostic group. Then, differences in brain volumes (within a set of predetermined brain regions implicated by previous studies^28–32^, incorporating frontal, temporal, insula, hippocampus, and 3rd ventricle) were assessed, comparing SZH to CON, and SZH-SIB with CON-SIB. We included the 3rd ventricle, rather than lateral ventricles, as it has been found that 3rd ventricle enlargement but not lateral ventricle enlargement corelates with cognitive deficits in SZH^33^. Then, cluster assignment was added as an additional covariate in these comparisons, to determine the extent to which between-group differences in brain volumes could be accounted for by cognitive cluster assignment.

## Results

### Differences in cognitive performance between the clusters

**Fig. 1:**
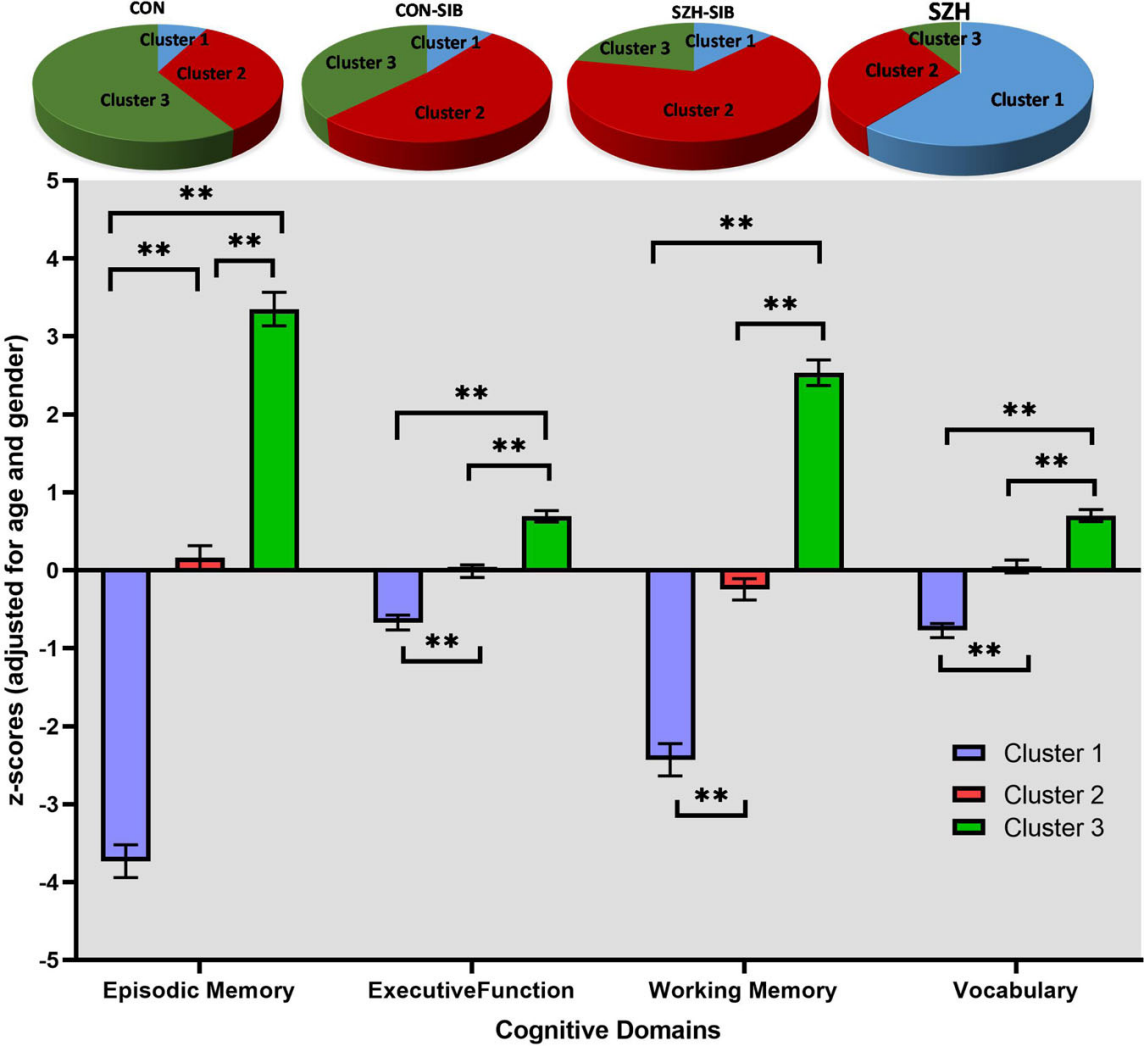
Group Differences in Cognitive Scores among Clusters. Controls (CON), their siblings (CON-SIB), schizophrenia patients (SZH), and their unaffected siblings (SZH-SIB). **Post hoc *p* < 0.01, *Post hoc *p* < 0.05.

We adopted a k-means clustering approach, using the four cognitive domain measures (adjusted for age and gender) across all participants irrespective of diagnostic group. Three cognitive function clusters were identified (Clusters 1–3). As shown in Fig. 1, the neurocognitive profiles indicated a ‘neuropsychologically normal’ cluster (Cluster 3, *N* = 88), a cognitively impaired cluster (Cluster 1, *N* = 78), and an intermediate cluster (Cluster 2, *N* = 108). To test for gender distribution differences across clusters, we performed a Pearson Chi-Square test. Results revealed no statistically significant differences in distribution, clusters 1, 2 and 3 comprised 50, 48, and 51 male participants respectively. ANOVAs assessed differences in cognitive performance between the clusters. Results showed a main effect of cluster assignment on all cognitive domains (*p* < 0.001). Post hoc analyses revealed that those in Cluster 1 had poorer performance in all cognitive domains, compared to those in Cluster 2 (*p* < 0.001) and Cluster 3 (*p* < 0.001). Cluster 2 also had poorer performance in all domains compared to Cluster 3 (*p* < 0.001). In follow-up analyses, all results remained unchanged after additionally adjusting for education (ANCOVAs); see supplementary material for full statistics.

Groups (SZH, SZH-SIB, CON, or CON-SIB) were not evenly distributed between the three clusters (Fig. 1 and Table 1, χ^2^ = 109.19, *p* < 0.001). SZH were mainly distributed to Cluster 1 (60.8%), followed by Cluster 2 (30.4%), and Cluster 3 (8.8%). SZH-SIB were mainly distributed to Cluster 2 (65.6%), followed by Cluster 3 (21.9%), and Cluster 1 (12.5%). CON-SIB were mainly distributed to Cluster 2 (52.1%), followed by Cluster 3 (37.5%), and Cluster 1 (10.4%). CON were mainly distributed to Cluster 3 (58.7%), followed by Cluster 2 (33.7%), and Cluster 1 (7.6%).

**Table 1:** Demographic characteristics of the sample by cluster groups, In all subjects, and in SZH only.

	In all subjects				
	Cluster 1	Cluster 2	Cluster 3	*P* values (*F* or *X*^*2*^)	Post Hoc
Variables	(*N* = 78)	(*N* = 108)	(*N* = 88)		
Diagnostic groups (CON/CON-SIB/SZH-SIB/SZH)	7/5/4/62	31/25/21/31	54/18/7/9	**3.01** **×** **10**^**−21**^ **(109.19)**^**b**^	–
Age (years)	30.6 ± 12.4	27.2 ± 12.1	29.6 ± 12.4	0.161 (1.84)^a^	–
Gender (male/female)	50/28	48/60	51/37	**0.021 (7.72)**^**b**^	–
Years of Schooling	11.3 ± 2.0	12.8 ± 2.3	14.9 ± 2.5	**9.40** **×** **10**^**−20**^ **(51.82)**^**a**^	1 < 2,3, 2 < 3

	In SZH only				
	Cluster 1	Cluster 2	Cluster 3	*P* values (*F* or *X*^*2*^)	Post Hoc
	(*N* = 62)	(*N* = 31)	(*N* = 9)		
Age (years)	32.1 ± 11.9	36.3 ± 14.1	34.8 ± 13.7	0.317 (1.16)^a^	–
Gender (male/female)	45/17	18/13	7/2	0.300 (2.41)^b^	–
Years of Schooling	11.4 ± 1.9	12.6 ± 2.2	13.8 ± 3.1	**0.001 (7.23)**^**a**^	1 < 2,3
CPZ-eq (mg/day)	395.3 ± 307.3	544.0 ± 534.0	344.5 ± 188.6	0.306 (1.21)	–
Age at onset (years)	20.1 ± 7.5	20.8 ± 7.4	26.7 ± 10.5	0.063 (2.84)	–
Duration of Illness	12.2 ± 1.6	15.5 ± 13.1	8.05 ± 9.55	0.240 (1.45)	–
SAPS	17.0 ± 13.6	16.7 ± 13.8	12.8 ± 11.5	0.676 (0.40)	–
SANS	23.9 ± 15.8	20.2 ± 13.5	15.4 ± 1.5	0.203 (1.62)	–

*CON* healthy controls, *CON-SIB* control-siblings, *SZH-SIB* schizophrenia-siblings, *SZH* schizophrenia, *CPZ-eq* chlorpromazine equivalents of total antipsychotics, *SAPS* Scale for the Assessment of Positive Symptoms, *SAN* Scale for the Assessment of Negative Symptoms.

Means ± SD are shown. *P* values < 0.05 are shown in boldface and *post hoc* analyses (Tukey) were performed.

^a^ANOVA.

^b^Pearson Chi-Square.

No difference in age was observed between clusters, but the gender balance and years of education were significantly different (see Table 1). Considering just the SZH in each cluster, the mean age, gender ratio, age at onset, duration of illness, chlorpromazine equivalents of total antipsychotics (CPZ-eq), and SAPS/SANS scores in SZH did not differ by cluster (*p* > 0.05). Years of education in SZH differed significantly by cluster (*p* < 0.05). SZH in Cluster 1 had lower years of education (*p* < 0.05).

### Differences in cognitive performance between the diagnostic groups

**Fig. 2:**
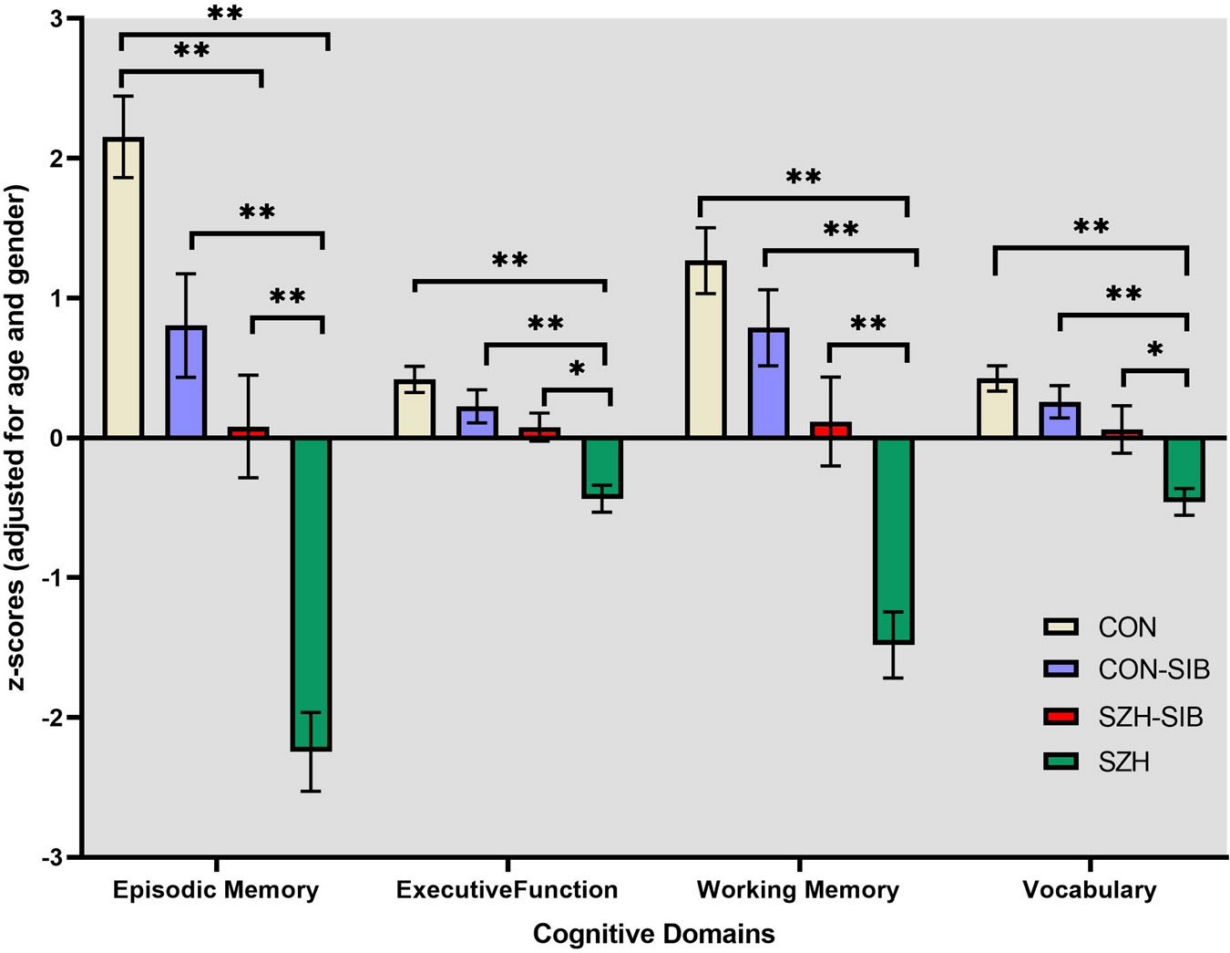
Cognitive domain score differences between Controls (CON), their siblings (CON-SIB), schizophrenia patients (SZH), and their unaffected siblings (SZH-SIB). **Post hoc *p* < 0.01, *Post hoc *p* < 0.05.

ANOVAs assessed group differences in cognitive performance between CON, CON-SIB, SZH-SIB, and SZH; see Fig. 2. Results revealed significant main effects of the group for all four (age and gender-adjusted) cognitive domain measures (*p* < 0.001). *Post hoc* analyses revealed that SZH had poorer performance in all four cognitive domains, differences were significant between SZH compared to SZH-SIB in executive function, working memory, vocabulary (*p* < 0.05), and episodic memory (*p* < 0.001). Between SZH and CON, and between SZH and CON-SIB, significantly poorer performance was seen in SZH in all four domains (*p* < 0.001). (See supplementary material for full statistics).

Compared to CON, SZH-SIB had significantly poorer episodic memory (*p* = 0.001) and working memory performance (at trend level, *p* = 0.054). Although there were no statistically significant differences between SZH-SIB and CON-SIB, all the effect sizes were negative (episodic memory, Cohen’s *d* = −0.41, *p* = 0.41; executive function, *d* = −0.21, *p* = 0.88; working memory; *d* = −0.36, *p* = 0.54, vocabulary, *d* = −0.22, *p* = 0.77) pointing to lower scores in SZH-SIB compared to CON-SIB. There were no statistically significant cognitive performance differences between CON-SIB and CON in any domain (see Fig. 2).

None of these results were seen to change after re-running the ANCOVAs with education as an additional covariate.

### Differences in ICV-Adjusted Volume between the diagnostic groups

Comparisons were conducted using ANCOVA with age and gender as covariates. Follow-up analyses then included cluster assignment as an additional covariate.

#### SZH vs CON

After FDR correction for multiple comparisons, smaller ICV-adjusted volumes were observed in SZH within bilateral DLPFC, right VLPFC, right superior temporal, bilateral inferior temporal, right middle temporal, and left insula compared to CON (*p* < 0.05). SZH had larger 3rd ventricle compared to CON (*p* < 0.05) (Table 2). SZH also had smaller left superior temporal volume (unadjusted *P* = 0.049), although this did not survive FDR correction. When cluster assignment was then added as an additional covariate, no significant differences were observed between SZH and CON in any ROIs (All FDR-corrected *p* values > 0.05) (Supplementary Table 1).

**Table 2:** Group Differences in ICV-Adjusted Volumes (age and gender as covariates).

	SZH vs CON				SZH-SIB vs CON-SIB				Pairwise Comparison	
	ANCOVA				ANCOVA				Significant Differences	
ICV Adjusted Volumes	F	p_unadjusted_	p_FDR_	η2	F	p_unadjusted_	p_FDR_	η2	SZH vs CON	SZH-SIB vs CON-SIB
Right Dorsolateral Prefrontal	8.610^a^	0.004	0.019	0.043	2.126	0.149	0.248	0.027	SZH < CON	–
Left Dorsolateral Prefrontal	7.111^a^	0.008	0.020	0.036	3.734	0.057	0.248	0.047	SZH < CON	–
Right Ventrolateral Prefrontal	7.084^a^	0.008	0.020	0.036	3.824	0.054	0.248	0.048	SZH < CON	–
Left Ventrolateral Prefrontal	2.216	0.138	0.173	0.012	2.614	0.110	0.248	0.033	–	–
Right Superior Temporal	5.192^a^	0.024	0.040	0.027	2.209	0.141	0.248	0.028	SZH < CON	–
Left Superior Temporal	3.941	0.049	0.074	0.020	1.522	0.221	0.276	0.020	SZH < CON	–
Right Inferior Temporal	9.900^a^	0.002	0.015	0.050	2.711	0.104	0.248	0.034	SZH < CON	–
Left Inferior Temporal	8.068^a^	0.005	0.019	0.041	2.157	0.146	0.248	0.028	SZH < CON	–
Right Middle Temporal	6.381^a^	0.012	0.023	0.032	2.837	0.096	0.248	0.036	SZH < CON	–
Left Middle Temporal	0.378	0.539	0.622	0.002	2.416	0.124	0.248	0.031	–	–
Right Insula	2.638	0.106	0.145	0.014	0.853	0.359	0.414	0.011	–	–
Left Insula	9.831^a^	0.002	0.015	0.049	0.685	0.411	0.440	0.009	SZH < CON	–
3rd Ventricle	6.788^a^	0.010	0.021	0.034	0.068	0.795	0.795	0.001	SZH > CON	–
Right Hippocampus	0.181	0.671	0.719	0.001	1.527	0.220	0.276	0.020	–	–
Left Hippocampus	0.041	0.841	0.841	0.000	1.858	0.177	0.266	0.024	–	–

^a^FDR corrected *p* < 0.05.

*CON* healthy controls, *CON-SIB* control-siblings, *SZH-SIB* schizophrenia-siblings, *SZH* schizophrenia.

#### SZH-SIB vs. CON-SIB

There were no statistically significant differences between SZH-SIB and CON-SIB in any ROIs (All FDR corrected p values > 0.05) (Table 2). When cluster assignment was then added as an additional covariate, still no significant differences were observed between SZH-SIB and CON-SIB in any ROIs (All FDR corrected *p* values > 0.05) (Supplementary Table 1).

### Differences in ICV-Adjusted Volume between the Clusters

Comparisons were conducted using ANCOVA with age and gender as covariates. Follow-up analyses then included diagnostic group as an additional covariate.

After FDR correction for multiple comparisons, larger ICV-adjusted volume was observed in Cluster 3 within right middle temporal compared to Cluster 1 and Cluster 2 (*p* < 0.05). Cluster 3 also had larger left middle temporal, bilateral inferior temporal and left insula compared to Cluster 1 and Cluster 2 although this did not survive FDR correction (All unadjusted *p* values < 0.05). Cluster 3 had smaller 3rd ventricle compared to Cluster 1 (unadjusted *P* = 0.013), but this did not survive FDR correction. Diagnosis (i.e., CON, CON-SIB, SZH-SIB, or SZH) was then added as an additional covariate. This abolished all between-cluster differences: even at an uncorrected threshold, no differences were observed between the clusters in any ROI (All uncorrected *p* values > 0.05) (Supplementary Table 2). As a follow-up analysis, we also examined brain volumetric differences between cluster groups for SZH only, using ANOVA. Results revealed that SZH within Cluster 1 (relatively intact group) have significantly larger right VLPFC volume compared to those within Cluster 2 and Cluster 3 (unadjusted *p* value = 0.041), however, this did not survive FDR correction. Differences in brain volumes between SZH in cluster 1 and cluster 2, assessed by ANCOVA, revealed no significant differences between clusters (all *p* values > 0.05) (Supplementary Table 4).

## Discussion

The current study used a clustering strategy to characterise cognitive subtypes within SZH and their unaffected siblings, investigate brain volume differences between diagnostic groups, and the extent to which cluster assignment could account for these. In all cognitive domains, SZH had poorer performance compared to CON and CON-SIB, in line with evidence that cognitive impairment is a core feature of schizophrenia affecting almost all cognitive domains, including episodic memory^34^, working memory^8^, and executive function^35^. SZH were also impaired relative to SZH-SIB, but with smaller effect sizes. Although no significant differences were seen between SZH-SIB and CON-SIB, across the domains understudy, all effect sizes were in the direction of poorer cognitive performance in SZH-SIB. Likewise, Ohi, et al.^15^ only found performance differences between first-degree relatives and controls on the attention subscale of the BACS (symbol coding), but effect sizes across other domains were in the direction of poorer performance in relatives. However, other studies have identified more significant impairments on neurocognitive tests in unaffected first‑degree relatives of SZH^36–39^. This supports the notion that cognitive impairment is a potential endophenotype of schizophrenia, reflecting a pathophysiological basis of vulnerability to schizophrenia rather than an epiphenomenon of the disease process^40,41^. Here, although the pattern of effect sizes alluded to impairments, none of the statistical comparisons showed any significant cognitive performance differences between SZH-SIB and CON-SIB in this sample. This might be due to their relatively young age; Barch, et al.^42^ found cognitive differences in SZH-SIB compared to CON were only detectable in those aged over 21, but not in younger SZH-SIB. Thus, it might be that robust differences only emerge at a slightly later age point than that of our SZH-SIB sample. Barch, et al.^42^ also concluded that the inconsistency between studies might be due to sampling differences in the unaffected relatives of SZH. Rather than siblings, most previous studies used less strict recruitment criteria: many included any first-degree relative, some included offspring, who could well be impacted by pre-or peri-natal care issues^41^.

Across the entire sample, we identified three distinct cognitive clusters based on age- and gender-adjusted cognitive domain scores. These clearly showed a ‘neuropsychologically normal’ cluster, a ‘cognitively impaired cluster’, and an ‘intermediate cluster’. As expected, the majority of SZH were assigned to the cognitively impaired cluster while most CON were assigned to the neuropsychologically normal cluster. Less than 10% of SZH were in the neuropsychologically normal cluster. This accords with previous studies showing that around 10% of SZH tend to be classed as neuropsychologically normal based on a clustering analysis^14–16^, although a handful of studies have reported higher percentages: for example, Allen, et al.^43^ found that 20% of SZH were classed as neuropsychologically normal in their sample. On the other hand, most SZH-SIB were placed in the intermediate cluster, providing evidence of some cognitive impairment affecting SZH-SIB. This tallies with the results of Ohi, et al.^15^ who also found most relatives to be assigned to the intermediate cluster. The intermediate cluster (Cluster 2) performed worse than previously reported scores in healthy individuals, implying some impairment was present. For example, in WMS-III logical memory, Cluster 2 (mean raw scores: LNS: 9.23, LM-1: 9.90, LM-2: 9.94) performed significantly worse than a large control group (*N* = 330 mean age of 36, LNS: 12.72) from Cosgrove, et al.^44^ and performed worse even compared to a healthy older control group (*N* = 107, mean age = 71, LM-1: 10.77, LM-2: 11.52) from Foley, et al.^45^ reported average scores of 10.77 and 11.52 for the WMS-III logical memory (1 and 2) subtests in a sample of 107 older controls aged between 55 and 93 (mean age = 70.97). In contrast, our Cluster 2 performed well below these (mean raw scores: LNS: 9.23, LM-1: 9.90, LM-2: 9.94), despite the scores from Foley et al. (all *p* values < 0.05). Thus, Cluster 2 appears to be intermediately, albeit significantly, impaired.

As expected, significantly smaller brain volumes within many of the ROIs were evident in SZH, in bilateral DLPFC, right VLPFC, right middle and superior temporal, bilateral inferior temporal, and left insula, compared to CON. These results accord with the literature, showing widespread reduced gray matter volume across frontal and temporal brain regions in SZH^46,47^. In SZH, pronounced gray matter alterations are detectable at the first episode, and are progressive; this has been attributed to processes, including neuroinflammation^48^, and cumulative antipsychotic exposure^49^. We also observed larger third ventricles in SZH compared to CON. The ventricular expansion was one of the earliest macroscopic brain structural abnormalities identified in SZH, studies have consistently linked it to volume loss in local cortical/subcortical structures^50,51^.

Then, we added cognitive cluster as an additional covariate to these analyses. This was seen to abolish all observed volumetric differences between SZH and CON. This highlights the importance of patients’ cognitive status in relation to the brain volume differences associated with schizophrenia: it seems that once cognitive status is accounted for, these differences are significantly diminished. Thus, brain volume reductions in SZH seem to be intimately tied to individual differences in cognitive performance levels, and therefore studies that aim to better characterise brain volume differences in SZH would be advised to consider cognitive performance in addition to the more typical measures of positive and negative symptom severity. Likewise, Ohi, et al.^15^ also found that adding cognitive cluster as a covariate significantly diminished the brain volume differences between their SZH, first-degree relative, and control samples, although insula and frontal differences remained significant. However it should be noted that Ohi, et al.^15^ used volume-based morphometry rather than the Freesurfer-based ROI approach used here, and their sample characteristics differed in that they included any first degree relatives (rather than just siblings); this group was significantly older than both their SZH and CON groups, and their CON group was only 25 in number. Nevertheless, both the current study and that of Ohi, et al.^15^ observed that adding diagnostic group as an additional covariate completely abolished the effects of cognitive cluster on regional volumes. This important finding underlines differences in cognitive profiles as a potent explanatory factor for the abnormalities in brain anatomy seen in schizophrenia. There is considerable heterogeneity in the literature around these abnormalities; the contribution of cognitive performance differences is underappreciated and often unaccounted for in these studies: the current finding supports a recommendation that comprehensive cognitive testing be included in all structural investigations going forward, to allow these effects to be accounted for and add clarity to the field.

No volumetric differences were seen between SZH-SIB and CON-SIB. This is in contrast with previous studies reporting frontotemporal gray matter reductions in unaffected relatives of SZH versus controls^52,53^, including in inferior and medial frontal cortex^54–56^, and in inferior and superior temporal gyrus^57^, suggesting a genetic contribution. We did not detect differences, and this could be attributed to sampling differences. In our study, we included only unaffected siblings of SZH as relatives. By contrast, some of the aforementioned studies also recruited offspring of SZH^54,57^ and those with a history of Axis 1 diagnoses (i.e. mood disorders)^53^; some studies have also contrasted with a control group at low genetic risk for schizophrenia^52^. However, familial and genetic bases of structural alterations should be interpreted with caution. Even though some studies have reported frontotemporal volume reduction in relatives, there are also some longitudinal studies showing these reductions are most evident in early adolescence and become less marked thereafter^58,59^.

The strengths of the current study include better size samples compared to similar previous work, and the inclusion of siblings only, in the unaffected relatives group. Previous studies have often included any first-degree relatives and thus the current sample is more homogenous; the inclusion of age- and gender-matched control siblings as a comparison group helps strengthen confidence in the inferences. However, the current study considered only a limited set of cognitive domains: other cognitive domains that are known to be impaired in SZH such as motor speed, verbal fluency, and social cognition should be investigated in future studies, to determine whether the current findings are generalisable to these. Another limitation is that the SZH sample predominantly consisted of males which is a common issue amongst SZH study samples^60,61^. Moreover, SZH had lower level of education compared to CON. Although we adjusted all analyses for education, this might have affected results. However, unmatched education level is also a common issue in the SZH literature^62,63^.

In conclusion, this study provides further insight into the cognitive heterogeneity of SZH, an age- and gender-matched control group, and their siblings. Unlike most previous investigations, groups were well-matched on demographic variables, only siblings were included as relatives, and brain volume measures were considered. Importantly, the observed brain volume differences between SZH and controls disappeared after adjustment for cluster assignment. This shows how closely linked cognitive performance levels are to regional brain volume differences in SZH, and points to the importance of accounting for heterogeneity in cognitive performance within SZH populations by incorporating a comprehensive battery of cognitive measures alongside brain imaging. Previous work has often neglected to do this, and this could contribute to discrepancies and inconsistencies in the literature: such an approach would allow better characterisation of the brain structural abnormalities associated with the disease.

## Methods

### Subjects

The present study includes data for 102 SZH (32 females, aged between 17 and 61, mean age 33.6), age- and gender-matched 92 CON (41 females, aged between 14 and 66, mean age 30.7), 48 CON-SIB (34 females, aged between 15 and 28, mean age 20.3), and 32 SZH-SIB (18 females, aged between 14 and 28, mean age 21.8, see Table 3). The data for subjects were obtained from the publicly available Northwestern University Schizophrenia Data Sharing for SchizConnect (NUSDAST) database^64^ and downloaded from http://schizconnect.org website. The Scale for the Assessment of Positive Symptoms (SAPS)^65^ and Scale for the Assessment of Negative Symptoms (SANS)^66^ were used to assess symptom severity. All patients were stabilized on antipsychotics for at least two weeks prior to the study^67^ and doses of medications were converted to chlorpromazine equivalents (see Table 3). The research centre defined exclusion criteria as having an intellectual disability based on DSM-IV, having a severe medical disorder or head injury, and having met the criteria for substance use based on DSM- IV, and written informed consent was obtained from all participants before participation^67^. More information on data sampling and recruitment has been described elsewhere^64,68^.

**Table 3:** Demographic characteristics of the sample by diagnostic groups.

	CON	CON-SIB	SZH-SIB	SZH	*P* values (*F* or *X*^*2*^)	Post Hoc
Variables	(*N* = 92)	(*N* = 48)	(*N* = 32)	(*N* = 102)		
Age	30.7 ± 13.3	20.3 ± 3.5	21.8 ± 3.4	33.6 ± 12.8	**5.06** **×** **10**^**−12**^ **(20.55)**^**a**^	SZH > SZH-SIB, CON-SIB, CON > SZH-SIB, CON-SIB
Gender (male/female)	51/41	14/34	14/18	70/32	**6.1** **×** **10**^**−5**^ **(22.14)**^**b**^	–
Years of Schooling	14.4 ± 2.7	12.9 ± 2.4	12.9 ± 2.7	12.0 ± 2.2	**6.1** **×** **10**^**−9**^**(14.79)**^**a**^	CON > SZH-SIB, SZH, CON-SIB
CPZ-eq (mg/day)	–	–	–	447.0 ± 404.2	–	–
Age at onset (years)	–	–	–	20.9 ± 7.9	–	–
Duration of Illness	–	–	–	12.8 ± 12.5	–	–
SAPS	–	–	–	16.6 ± 13.4	–	–
SANS	–	–	–	22.0 ± 15.0	–	–

*CON* healthy controls, *CON-SI* control-siblings, *SZH-SIB* schizophrenia-siblings, *SZH* schizophrenia, *CPZ-eq* chlorpromazine equivalents of total antipsychotics, *SAPS* Scale for the Assessment of Positive Symptoms, *SANS* Scale for the Assessment of Negative Symptoms.

Means ± SD are shown. *P* values < 0.05 are shown in boldface and *post hoc* analyses (Tukey) were performed.

^a^ANOVA.

^b^Pearson Chi-Square.

### MRI Acquisition

NUSDAST collected MRI scans with a Siemens 1.5 T Vision Scanner. The details of the acquisition process are described elsewhere^64,68^. Following parameters were defined by the research centre to collect high-resolution T1-weighted structural images using an MPRAGE sequence: TR = 9.7 ms, TE = 4 ms, flip = 10°, ACQ = 1, 256 × 256 matrix, 1 × 1 mm in-plane resolution, 128 slices, slice thickness 1.25 mm, 5:36 min scan time each^68^. All images were processed via FreeSurfer 3.0.4^69^ and the cortical parcellations were derived based on the Destrieux atlas^70^. Regional gray matter volumes were derived from the Destrieux parcellation. We calculated intra-cranial (ICV)-adjusted volumes by dividing the volume in each ROI by the total ICV and multiplying by 100 ((volume in ROI/ICV)*100). We then defined the frontal ROI volumes as follows: DLPFC (sum of superior frontal, and rostral and caudal middle frontal), VLPFC (sum of pars opercularis, pars orbitalis, and pars triangularis), OFC (sum of lateral and medial orbital frontal). The temporal ROIs were superior temporal, middle temporal, and inferior temporal. We also included insula volume and hippocampus volume. All the ROIs were assessed separately as left and right-sided.

### Cognitive Measures

All participants completed a neuropsychological battery testing executive function, episodic memory, working memory, and vocabulary. Tasks were as follows.

From the Wechsler Memory Scale—Third Edition (WMS-III). Digit Span (total forward and backwards): participants recite back a sequence of numbers in the same order, and in reverse order^71^. Spatial Span (total forward and backwards): participants are asked to repeat a spatial sequence demonstrated by examiner in the same order (forward) and reverse order (backward)^72^. Letter Number Sequencing: participants are asked to repeat a mixed list of letters and numbers in alphabetic and ascending orders^72^. Logical Memory subtest: participants are asked to verbally recall a given story immediately (LM I, immediate version) and after a delay interval (LM II, delayed version)^72^, and Family Pictures subtest: subjects are shown a series of pictures of scenarios which participants are required to recall^72^.

Wisconsin Card Sorting Test (WCST)^73^: subjects sort 64 cards based on the colour, shape, and numbers, perseverative errors occur when a participant persists in using wrong rule despite negative feedback^74^.

Wechsler Adult Intelligence Scale^75^. Matrix Reasoning subtest: participants select images for pattern completion^76^. Vocabulary subtest: subjects define presented words^77^.

To create the following cognitive domains, we summed z-scores as follows (as recommended by NUSDAST researchers^78^):

- Working Memory: Sum of z-scores from Wechsler Memory Scale—Third Edition (WMS-III); including Digit Span (total forward and backwards), Spatial Span (total forward and backwards), and Letter-Number Sequencing
- Episodic Memory: WMS-III Logical Memory and Family Pictures subtests
- Executive Function: perseverative errors on the WCST and WAIS-III Matrix Reasoning subtest
- Vocabulary: scores from the Wechsler Adult Intelligence Scale Vocabulary subtest

Each domain score was converted to standardized z-scores using the mean and SDs of the whole sample and adjusted for age and gender by firstly fitting linear regression models to these (with age and gender as predictors), and then using the residuals from these linear regression models as the basis for subsequent analyses.

### ROI Approach

The ROIs focused on fronto-temporal regions since metanalyses have identified consistent volumetric reductions in these in SZH vs. CON. Frontal ROIs were dorsolateral prefrontal and ventrolateral prefrontal regions, the temporal ROIs were superior temporal, middle temporal, and inferior temporal^29,31,32^. We also included insula volume^30^ and hippocampus volume^28^ as these have also been shown to be reduced in SZH compared to CON. Finally, we included 3^rd^ ventricle volume which is consistently shown to be increased in SZH^29^.

### Statistical Analysis

Data were analysed using IBM SPSS Statistics 21.0. *P* values < 0.05 (two-tailed) were considered statistically significant. On demographic and age- and gender-corrected cognitive domains, groups (within diagnostic groups and clusters separately) were compared using ANOVAs (for age, years of schooling, and cognitive domains), and a χ^2^ test (for gender) and ANOVAs followed by Tukey’s post hoc tests to assess significant differences among the diagnostic groups and cluster groups. Cohen’s *d* was used to evaluate standardized effect sizes (https://lbecker.uccs.edu/). The possible effect of the diagnostic group on ICV-adjusted volumes was tested using ANCOVAs in which age and gender were included as covariates. SZH vs CON and SZH-SIB vs CON-SIB comparisons were conducted separately, to ensure results were not confounded by differences in age. This was important given strong ageing effects on brain volumes both in controls^79^ and schizophrenia^80^. To assess the extent to which between-groups differences could be accounted for by cognitive cluster assignment, a follow-up analysis included cluster assignment as an additional covariate. To assess differences in SZH brain volumes according to cluster assignment, we compared brain volumes of SZH in cluster 1 with SZH in cluster 2 using ANCOVA, with age and gender as covariates. Because of the small number of cluster 3 (*N* = 9) in SZH we did not include cluster 3 in this analysis as it is insufficient for ANCOVA.

Multiple comparisons correction was conducted using false discovery rate (FDR) with the Benjamini-Hochberg method^81^, and *p* < 0.05 was considered statistically significant. A k-means clustering approach was performed to identify distinct clusters in cognitive performance across all participants, based on all four of the cognitive domain Z-scores (adjusted for age and gender), and independent of diagnostic status. The k-means clustering partitioned the sample into clusters with each participant assigned to the cluster with the nearest mean; a k-means approach was selected so as to be parsimonious with most previous similar investigations, also it is more suitable for continuous variables than hierarchical clustering, which is better suited to categorical variables^82^. A 3-cluster solution was found to be optimal. A range of cluster solutions (2–5) was tested, and a 3-cluster solution resulted in the largest Gap statistic, optimality was also indicated based on the Elbow Curve. A 3-cluster solution accords with that employed by Ohi, et al.^15^, and the overwhelming majority of previous studies have also identified a 3-cluster solution^22^.

## Supplementary information

SUPPLEMENTAL MATERIAL

## Data Availability

The data that support the findings of this study are available from the corresponding author upon reasonable request.
