# Supplementary material for: Clustering of cognitive subtypes in schizophrenia patients and their siblings: relationship with regional brain volumes
Source: Schizophrenia (Heidelb). 2022 May 9;8(1):50. doi: 10.1038/s41537-022-00242-y (PMC9261107; doi:10.1038/s41537-022-00242-y)
Supplement: Supplementary file 1 — SUPPLEMENTAL MATERIAL [file 41537_2022_242_MOESM1_ESM.docx]

**Clustering of cognitive subtypes in schizophrenia patients and their siblings: relationship with regional brain volumes**

**Supplementary Material**

**Details of Statistics on Differences in cognitive performance among the diagnostic groups**

Results revealed significant main effects of group for all four cognitive domain measures (adjusted for age and gender) derived from the test battery (Fig 1, episodic memory, *F*_3,273_ = 45.3, *P* = 9.57 × 10^-24^; executive function, *F*_3,273_ = 16.18, *P* = 1.05 × 10^-9^; working memory, *F*_3,273_ = 27.8, *P* = 1.09 × 10^-15^ and vocabulary, *F*_3,273_ = 16.8, *P* = 5.01 × 10^-10^). *Post hoc*

analyses revealed that SZH had poorer performance in all cognitive domains compared to other groups (SZH vs SZH-SIB: episodic memory, Cohen's *d* = − 0.94, *P* = 1.7 × 10^−^ ^4^; executive function, *d* = − 0.64, *P* = 0.024; working memory, *d* = − 0.76, *P* = 0.002 and vocabulary, *d* = − 0.54, *P* = 0.026, SZH vs CON-SIB: episodic memory, *d* = − 1.21, *P* = 1.7 × 10^−10^; executive function, *d* = − 0.73, *P* = 1.5 × 10^-4^; working memory, *d* = − 1.06, *P* = 6.4 × 10^-8^ and vocabulary, *d* = − 0.81, *P* = 5.4×10^-5^, SZH vs CON: episodic memory, *d* = − 1.56, *P*

= 4.7 × 10^−13^; executive function, *d* = − 0.91, *P* = 6.3 × 10^-10^; working memory, *d* = − 1.19, *P*

= 4.7 × 10^-13^ and vocabulary, *d* = − 0.97, *P* = 4.2×10^-10^).

**Details of Statistics on Differences in cognitive performance among the clusters**

Results showed a main effect of cluster assignment on all cognitive domains (Fig 2, episodic memory, *F*_2,273_ = 317.7, *P* = 1.04 × 10^-71^; executive function, *F*_2,273_ = 60.9, *P* = 1.35 × 10^-22^; working memory, *F*_2,273_ = 208.1, *P* = 1.75 × 10^-55^ and vocabulary, *F*_2,273_ = 70.4, *P* = 2.38 × 10^-^ ^25^). *Post hoc* analyses revealed that Cluster 1 had poorer performance in all cognitive domains compared to other clusters (Cluster 1 vs Cluster 2: episodic memory, Cohen's *d* = − 2.26, *P* = 5.1 × 10^−^ ^9^; executive function, *d* = − 0.79, *P* = 1.81 × 10^−^ ^7^; working

memory, *d* = − 1.34, *P* = 5.1 × 10^−^ ^9^ and vocabulary, *d* = − 0.99, 5.2 × 10^−^ ^9^; Cluster 1 vs

Cluster 3: episodic memory, *d* = − 3.66, *P* = 5.1 × 10^−^ ^9^; executive function,

*d* = − 0.64, *P* = 5.1 × 10^−^ ^9^; working memory, *d* = − 2.95, *P* = 5.1 × 10^−^ ^9^ and vocabulary, *d* = − 1.93, *P* = 5.1 × 10^−^ ^9^). Cluster 2 also had poorer performance on all domains compared to Cluster 3 (episodic memory, *d* = − 1.38, *P* = 5.1 × 10^−^ ^9^; executive function, *d* = −0.91, *P* = 1.2 × 10^−^ ^8^; working memory, *d* = − 1.88, *P* = 5.1 × 10^−^ ^9^ and vocabulary, *d* = − 0.82, *P* = 1.1 × 10^−^ ^7^). All results remained significant after adjusting for education (ANCOVAs).

**Details of Statistics on Differences in ICV-adjusted Volumes between SZH and CON**

After FDR correction for multiple comparisons, smaller ICV-adjusted volumes were observed in SZH within bilateral DLPFC (right: Cohen’s *d*= -0.50, *F*_1,190_=8.61, *P*=0.019; left: *d*= -0.48, *F*_1,190_=7.11, *P*=0.020) right VLPFC (*d*= -0.48, *F*_1,190_=7.08, *P*=0.020), right superior

temporal (*d*= -0.45, *F*_1,190_=5.19, *P*=0.040), bilateral inferior temporal (right: *d*= -0.51, *F*_1,190_=9.90, *P*=0.015; left: *d*= -0.49, *F*_1,190_=8.07, *P*=0.019), right middle temporal (*d*= -0.42, *F*_1,190_=6.38, *P*=0.023), and left insula (*d*= -0.54, *F*_1,190_=9.83, *P*=0.015) compared to CON. SZH had larger 3^rd^ ventricle (*d*= 0.47, *F*_1,190_=6.79, *P*=0.021) compared to CON.

**Supplementary Table 1**

Group Differences in ICV-Adjusted Volumes between cluster groups (age and gender as covariates)

**Between-cluster Differences Pairwise Comparison ANCOVA Significant Differences**

| **ICV Adjusted Volumes** | F | punadjusted | pFDR | η2 |  |
| --- | --- | --- | --- | --- | --- |
| Right Dorsolateral Prefrontal | 2.971 | .053 | .111 | .022 | - |
| Left Dorsolateral Prefrontal | 2.853 | .059 | .111 | .021 | - |
| Right Ventrolateral Prefrontal | 2.154 | .118 | .197 | .016 | - |
| Left Ventrolateral Prefrontal | 1.272 | .282 | .302 | .009 | - |
| Right Superior Temporal | 1.449 | .237 | .278 | .011 | - |
| Left Superior Temporal | 1.931 | .147 | .217 | .014 | - |
| Right Inferior Temporal | 3.988 | .020 | .060 | .029 | - |
| Left Inferior Temporal | 4.858 | .008 | .056 | .035 | - |
| Right Middle Temporal | 7.361* | .001 | .015 | .052 | 1<3, 2<3 |
| Left Middle Temporal | 4.265 | .015 | .056 | .031 | - |
| Right Insula | .659 | .518 | .518 | .005 | - |
| Left Insula | 3.592 | .029 | .073 | .026 | - |
| 3^rd^ Ventricle | 4.430 | .013 | .056 | .032 | - |
| Right Hippocampus | 1.854 | .159 | .217 | .014 | - |
| Left Hippocampus | 1.431 | .241 | .278 | .011 | - |

*FDR corrected p<.05

**Supplementary Table 2**

Group Differences in ICV-Adjusted Volumes (age, gender, and cluster as covariates)

**SZH vs CON SZH-SIB vs CON-SIB ANCOVA ANCOVA**

| **ICV Adjusted Volumes** | F | punadjusted | pFDR | η2 | F | punadjusted | pFDR | η2 |
| --- | --- | --- | --- | --- | --- | --- | --- | --- |
| Right Dorsolateral Prefrontal | 3.309 | .071 | .225 | .017 | 1.845 | .178 | .310 | .024 |
| Left Dorsolateral Prefrontal | 3.102 | .080 | .225 | .016 | 3.168 | .079 | .310 | .041 |
| Right Ventrolateral Prefrontal | 3.135 | .078 | .225 | .016 | 3.627 | .061 | .310 | .046 |
| Left Ventrolateral Prefrontal | .418 | .519 | .580 | .002 | 2.826 | .097 | .310 | .036 |
| Right Superior Temporal | 2.112 | .148 | .278 | .011 | 2.268 | .136 | .310 | .029 |
| Left Superior Temporal | 1.090 | .298 | .447 | .006 | 1.523 | .221 | .310 | .020 |
| Right Inferior Temporal | 2.938 | .088 | .225 | .015 | 2.640 | .108 | .310 | .034 |
| Left Inferior Temporal | 2.243 | .136 | .278 | .012 | 1.610 | .208 | .310 | .021 |
| Right Middle Temporal | .688 | .408 | .510 | .004 | 1.849 | .178 | .310 | .024 |
| Left Middle Temporal | .101 | .750 | .750 | .001 | 1.736 | .192 | .310 | .023 |
| Right Insula | 2.912 | .090 | .225 | .015 | .963 | .330 | .381 | .013 |
| Left Insula | 3.098 | .080 | .225 | .016 | .588 | .445 | .477 | .008 |
| 3^rd^ Ventricle | .723 | .396 | .510 | .004 | .080 | .778 | .778 | .001 |
| Right Hippocampus | 1.140 | .287 | .447 | .006 | 1.269 | .264 | .330 | .017 |
| Left Hippocampus | .375 | .541 | .580 | .002 | 1.483 | .227 | .310 | .019 |

*Abbreviations*: CON, healthy controls; CON-SIB, control-siblings; SZH-SIB, schizophrenia-siblings; SZH, schizophrenia

**Supplementary Table 3**

Group Differences in ICV-Adjusted Volumes between cluster groups (age, gender, and diagnosis as covariates)

**ANCOVA**

| **ICV Adjusted Volumes** | F | punadjusted | pFDR | η2 |
| --- | --- | --- | --- | --- |
| Right Dorsolateral Prefrontal | .933 | .395 | .599 | .007 |
| Left Dorsolateral Prefrontal | .493 | .612 | .656 | .004 |
| Right Ventrolateral Prefrontal | .039 | .962 | .962 | .000 |
| Left Ventrolateral Prefrontal | .739 | .479 | .599 | .005 |
| Right Superior Temporal | .564 | .570 | .656 | .004 |
| Left Superior Temporal | .796 | .452 | .599 | .006 |
| Right Inferior Temporal | 1.103 | .333 | .599 | .008 |
| Left Inferior Temporal | 1.364 | .257 | .599 | .010 |
| Right Middle Temporal | 2.415 | .091 | .455 | .018 |
| Left Middle Temporal | 2.670 | .071 | .455 | .020 |
| Right Insula | 1.119 | .328 | .599 | .008 |
| Left Insula | 1.272 | .282 | .599 | .009 |
| 3^rd^ Ventricle | 2.995 | .052 | .455 | .022 |
| Right Hippocampus | 1.294 | .276 | .599 | .010 |
| Left Hippocampus | .829 | .438 | .599 | .006 |

**Supplementary Table 4**

Group Differences in ICV-Adjusted Volumes between cluster 1 and cluster 2 within SZH (age, gender, and diagnosis as covariates)

**ANCOVA**

| **ICV Adjusted Volumes** | F | punadjusted | pFDR | η2 |
| --- | --- | --- | --- | --- |
| Right Dorsolateral Prefrontal | 0.093 | 0.761 | 1.000 | 0.001 |
| Left Dorsolateral Prefrontal | 0.380 | 0.539 | 1.000 | 0.004 |
| Right Ventrolateral Prefrontal | 2.565 | 0.113 | 0.848 | 0.028 |
| Left Ventrolateral Prefrontal | 0.017 | 0.895 | 1.000 | 0.000 |
| Right Superior Temporal | 0.038 | 0.847 | 1.000 | 0.000 |
| Left Superior Temporal | 0.000 | 0.992 | 1.000 | 0.000 |
| Right Inferior Temporal | 0.073 | 0.788 | 1.000 | 0.001 |
| Left Inferior Temporal | 0.079 | 0.779 | 1.000 | 0.001 |
| Right Middle Temporal | 0.000 | 1.000 | 1.000 | 0.000 |
| Left Middle Temporal | 2.702 | 0.104 | 0.848 | 0.029 |
| Right Insula | 0.001 | 0.973 | 1.000 | 0.000 |
| Left Insula | 0.165 | 0.686 | 1.000 | 0.002 |
| 3^rd^ Ventricle | 0.003 | 0.958 | 1.000 | 0.000 |
| Right Hippocampus | 0.813 | 0.370 | 1.000 | 0.009 |
| Left Hippocampus | 0.161 | 0.689 | 1.000 | 0.002 |
